# A Compact Low-Power LoRa IoT Sensor Node with Extended Dynamic Range for Channel Measurements

**DOI:** 10.3390/s18072137

**Published:** 2018-07-03

**Authors:** Thomas Ameloot, Patrick Van Torre, Hendrik Rogier

**Affiliations:** Department of Information Technology (INTEC), Ghent University/IMEC, Technologiepark-Zwijnaarde 15, B-9052 Ghent, Belgium; patrick.vantorre@ugent.be (P.V.T.); hendrik.rogier@ugent.be (H.R.)

**Keywords:** wireless sensor networks, Internet of Things, LoRa, radiowave propagation

## Abstract

As sub-GHz wireless Internet of Things (IoT) sensor networks set the stage for long-range, low-data-rate communication, wireless technologies such as LoRa and SigFox receive a lot of attention. They aim to offer a reliable means of communication for an extensive amount of monitoring and management applications. Recently, several studies have been conducted on their performance, but none of these feature a high dynamic range in terms of channel measurement. In this contribution an autonomous, low-power, LoRa-compatible wireless sensor node is presented. The main uses for this node are situated in LoRa channel characterization and link performance analysis. By applying stepped attenuators controlled by a dynamic attenuation adjustment algorithm, this node provides a dynamic range that is significantly larger than what is provided by commercially available LoRa modules. The node was calibrated in order to obtain accurate measurements of the received signal power in dBm. In this paper, both the hardware design as well as some verification measurements are discussed, unveiling various LoRa-related research applications and opportunities.

## 1. Introduction

Wireless sensor networks (WSNs) are at the heart of the ever-developing Internet of Things (IoT). They have received a great deal of attention across a diverse range of research areas such as environmental monitoring, disaster surveillance, smart buildings, smart grids, healthcare, agricultural control, predictive maintenance, transport and asset tracking [1,2,3,4]. Seeing WSN deployment over larger and larger areas, it becomes evident to consider the use of sub-GHz frequency bands for communication in these long-range networks, given the superior propagation characteristics when compared to higher frequency bands. Conveniently, the reduction of the available bandwidth at lower frequencies does not degrade performance since communication links in WSNs are typically very low data-rate connections anyway.

Multiple standards for low data-rate sub-GHz communication have been proposed, the most prominent of which are LoRa, SigFox and Dash7 [1,5,6,7,8]. As Dash7 is promoted to be a medium-range technology [7], for long-range networks, the scope has shifted towards LoRa and SigFox. The adjustable data rate of the LoRa standard, combined with its slightly higher level of global coverage [5,6] makes for a very promising technology both for applications in WSNs and other long-range, low data-rate connections. LoRa is based on a chirp spread spectrum (CSS) modulation technology. To encode information, this technology uses wideband frequency modulated pulses called chirps to achieve spreading gain, which results in the successful reception of packets at extremely low signal-to-noise ratio (SNR) levels. The LoRa modulation technique was recently described in a rigorous way by L. Vangelista [9]. Additionally, the expected performance of LoRa has been discussed in a decent number of other publications [8,10,11,12,13], all of them praising the potential of LoRa modulation, but some also warning for reduced performance as the number of end-devices grow [8,12,13].

To explore the capabilities of LoRa modulation, a suitable radio platform is needed. A commercial LoRa platform frequently used in literature is the SX127X family from Semtech Corp [8,14,15,16,17,18]. Unfortunately, as most commercially available LoRa modules are tailored towards applications, they often lack dedicated measurement functions required for research applications. On the other end of the spectrum, lab-grade software radios do offer a lot of functionality, but in addition to being very expensive, they can also be very bulky for certain types of research. In fact, in any type of studies involving mobility or areas that are difficult to reach, both the size and power dependency of research equipment can seriously impede the possible research applications. The middle ground can be found by integrating a commercially available LoRa module into a system with more capabilities to create a compact, low-power wireless sensor node.

In this paper, such a node is proposed, designed and tested. For LoRa compatibility, a commercially available LoRa transceiver module—the RN2483 (by Microchip Technology Inc., Chandler, AZ, USA)—is used. Being built around a Semtech SX1276 transceiver (by Semtech, Camarillo, CA, USA), this module offers LoRa modulation for communication in both the 434 MHz and 868 MHz industrial, scientific and medical (ISM) radio bands. The RN2483 also offers channel measurement functions, such as SNR measurement, but due to the fact that LoRa is a proprietary technology, research determining the capabilities of this technology is bound to the capabilities of the most sensitive transceiver available, in this case: the SX1276. In the sensor node proposed in this paper, this is partly circumvented by using stepped attenuators to considerably increase the dynamic range of the measurements. The transceiver module is also certified to the LoRaWAN 1.0 specification, enabling the use of this node within the existing LoRaWAN network infrastructure [19].

In Section 2, the hardware design and calibration of the wireless sensor node is described in detail. To verify the correct operation of the node, several experiments were carried out in and around the city of Ghent, Belgium. The setup for these measurements is described in Section 3 and the results of these measurements are discussed in Section 4. Finally, in Section 5, a conclusion to this work is presented.

## 2. Hardware Design

**Figure 1:**
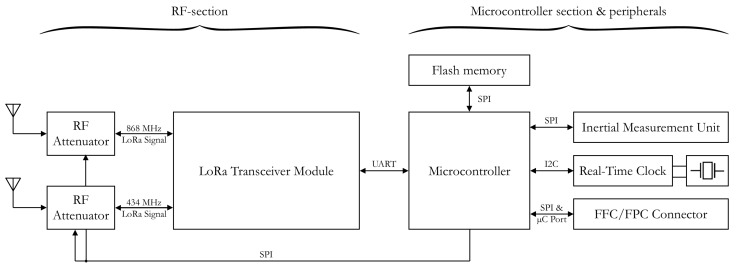
Block diagram of the wireless sensor node.

To give a full overview of the hardware design, a block diagram of the node is presented in Figure 1. The largest blocks in this diagram depict an 8-bit, low-power microcontroller—a C8051F342 (by Silicon Labs, Austin, TX, USA)—and the earlier mentioned RN2483 LoRa transceiver module. These components provide the core functionality of the microcontroller section and the RF-section, respectively.

### 2.1. Microcontroller Section

The C8051F342 is a general purpose microcontroller featuring a high-speed pipelined 8-bit microcontroller core, 64 kB of flash memory, two timers, a USB function controller and multiple popular bus interfaces such as System Management Bus (SMBus), Inter-Integrated Circuit (I^2^C), Universal Asynchronous Receiver-Transmitter (UART) and Enhanced Serial Peripheral Interface (SPI). Most of these interfaces can be routed through any of the available I/O Ports using the on-chip I/O-configurator, yielding a versatile, yet compact, low-cost and low-power control center for the wireless sensor node. The microcontroller can be programmed and debugged through the integrated two-wire C2 debug interface using a Silicon Labs USB Debug Adapter.

### 2.2. RF-Section

Among other things, the LoRa transceiver module is equipped with a LoRaWAN Protocol Stack, a 434 MHz and 868 MHz LoRa Technology Radio and a command processor providing the user with an easy-to-use ASCII-over-UART command interface. According to the transceiver’s documentation, the module can theoretically achieve receiver sensitivities down to −146 dBm by selecting the appropriate spreading factor (SF) and receiver bandwidth (RBW) of the LoRa modulation scheme [5]. Complemented by the integrated 14 dB power amplifier, very high link budgets can be obtained. In addition to LoRa modulation, the transceiver module also features Frequency Shift Keying (FSK) and Gaussian Frequency Shift Keying (GFSK) modulation [5].

With regard to channel characterization and link performance, the RN2483 transceiver can provide the user with the SNR of the last received packet. Unfortunately, no metrics on the measurement scheme used for this are given in the transceiver’s documentation. Therefore a calibration procedure was carried out in an anechoic chamber, linking the measured SNR value to the received power, thus establishing a baseline for SNR measurements.

**Figure 2:**
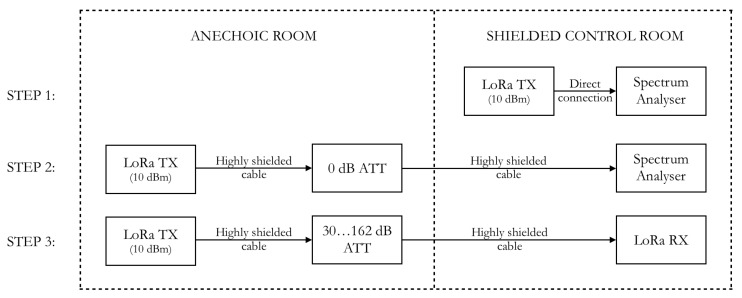
Calibration setup.

The calibration method outlined in Figure 2 is performed in three phases. In the first phase, the LoRa transmitter (TX) is connected directly to a Rohde and Schwartz FSV40 spectrum analyser, to accurately measure the transmitted power. Second, the TX is placed in the anechoic chamber and is connected via low-leakage coaxial transmission lines and a high-precision stepped attenuator to the spectrum analyzer in the shielded control room. In this step, the losses induced by the coaxial cables are measured to be calibrated away. The setup with the TX in the anechoic chamber and the RX in the shielded control room is necessary to provide enough isolation for the third measurement phase, where the attenuation has to be increased to over 150 dB in order to reach the detection limit of the LoRa RX.

**Figure 3:**
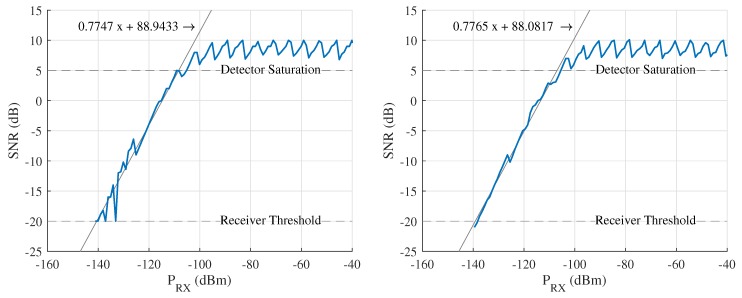
SNR measurement calibration for 434 MHz (**left**) and 868 MHz (**right**).

In this third phase, using the stepped attenuator, the attenuation is adjusted from 50 dB to 162 dB, which is the maximum setting. For every data point in the calibration, ten SNR measurements were performed and averaged. This procedure was performed for both the 434 MHz and 868 MHz frequency bands. The results of the calibration measurements are shown in Figure 3.

The calibration data show that using its default settings (SF = 12 and RBW = 125 kHz), the transceiver achieves a receiver sensitivity of −140 dBm, which corresponds to an SNR of −20 dB. This sensitivity is slightly better than the value of −136 dBm specified by the manufacturer for the default parameters. Note for measured SNR values greater than 5, the detector is seen to saturate and behave in a highly non-linear fashion. In the linear region, first-order regression models are fitted to the calibration data, yielding the following formula to calculate the received power ($P_{\mathrm{RX},\mathrm{dBm}}$) based on the measured SNR (${\mathrm{SNR}}_{\mathrm{meas},\mathrm{dB}}$):

(1) $$P_{\mathrm{RX},\mathrm{dBm}}=\frac{{\mathrm{SNR}}_{\mathrm{meas},\mathrm{dB}}-88.51}{0.7756}$$

Because of the strong similarity of the models for both frequency bands (their difference is lower than the measurement resolution), it is concluded that this relation is valid for both bands. Furthermore, the coefficients found for these models are very similar to those of the model presented for 868 MHz in [20].

As can be seen in Figure 3, only a relatively small SNR detection range is actually available. To resolve this issue, the RF-section also features two stepped attenuators. These attenuators serve to increase the range of the signal level measurement by dynamically attenuating signals that are too strong. The measured received power is described by

(2) $$P_{\mathrm{RX},\mathrm{dBm}}=P_{\mathrm{ANT},\mathrm{dBm}}-{\mathrm{ATT}}_{\mathrm{dB}}$$

with ${\mathrm{ATT}}_{\mathrm{dB}}\geq 0$ and $P_{\mathrm{ANT},\mathrm{dBm}}$ equal to the received power at a well-matched antenna, neglecting the minimal losses of the short transmission lines.

This is rewritten to find the received power at the antenna:

(3) $$P_{\mathrm{ANT},\mathrm{dBm}}=P_{\mathrm{RX},\mathrm{dBm}}+{\mathrm{ATT}}_{\mathrm{dB}}.$$

**Figure 4:**
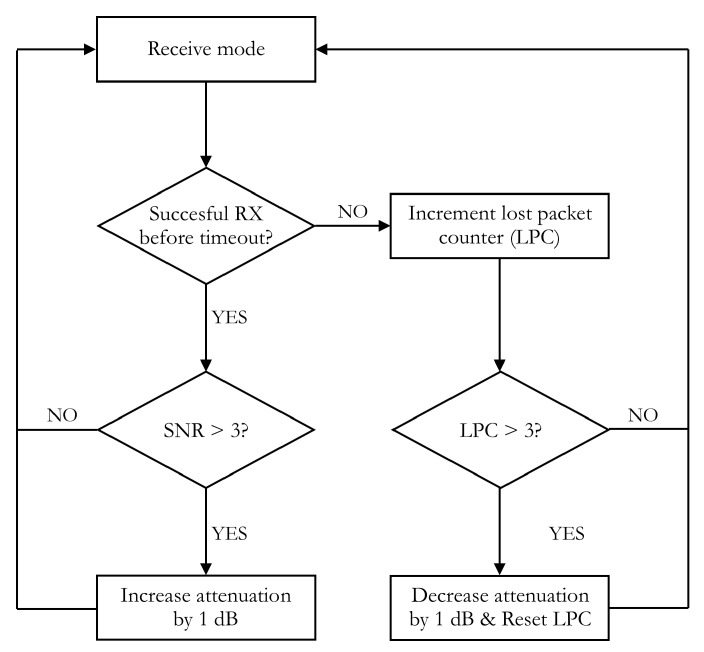
Flowchart of the attenuation adjustment algorithm.

**Attenuation levels can vary between** 0 dB and 31.5 dB and can be changed in 0.5 dB steps using an SPI bus. The pertinent attenuation adjustment algorithm is visualized in the flowchart depicted in Figure 4. In short, this algorithm either increases the attenuation by 1 dB when the SNRs of the received packets approach the saturation level of the detector—which is approximately equal to 5 dB, so a threshold of 3 dB is chosen—or lowers the attenuation by 1 dB when three subsequent packets are no longer received. The amount of lost packets needed to cause a decrease of attenuation was chosen at three because a lower value may cause the system to react to sudden changes in SNR caused by momentaneous interference issues while a higher value may cause the system to lose too much packets before adapting to a real change in propagation conditions. The step size was chosen at 1 dB because using the full 0.5 dB resolution of the attenuators would cause the amount of time needed for the algorithm to settle to the appropriate settings to be too high given that the amount of packets that can be sent per minute is subject to quite severe duty cycle regulations. On the other hand, using an even larger step size would result in a more sizable loss of resolution, which is also undesirable.

Given that the LoRa module has a linear SNR measurement range corresponding to a signal level range of 32 dB, a simple calculation reveals that the use of attenuators at the receiver’s side of the link increases the node’s theoretical dynamic range for signal measurements to 63.5 dB. Furthermore, by using the attenuators of the transmitter as well, this dynamic range can be shifted over an additional 31.5 dB. However, since this affects the transmitted signal power, receivers located further away from the transmitter could experience an increase in packet loss when this strategy is used. Consequently, care should be taken when shifting the dynamic range when multiple receivers are used. In addition to received power measurements, more straightforward link statistics such as packet loss can be gathered by using the general purpose microcontroller described earlier.

### 2.3. Peripherals

To diversify the potential applications of the wireless node, some peripherals are added to the microcontroller section of the design. First, a 32 Mbit flash memory IC is added to significantly enhance storage space on the node, thus allowing prolonged measurement campaigns to take place. Second, an inertial measurement unit (IMU) is integrated into the system. This sensor encompasses a 3D accelerometer, a 3D gyroscope and a 3D magnetometer, adding applications such as indoor navigation and motion monitoring to the list of possible use cases. Both the flash memory and the IMU can be accessed by means of the microcontroller’s SPI interface. The third addition to the node is a real-time clock (RTC) with its own separate 32.768 kHz quartz crystal for keeping time, which is exploited to set timestamps and to wake up the system out of low-power mode using interrupts. Especially this last feature may considerably reduce the total energy consumption of the node, which is very beneficial when using a battery as power source. The microcontroller can communicate with the RTC using its I^2^C interface and offers a dedicated connection for interrupts. Finally, through a flat flexible cable (FFC) and flat printed circuit cable (FPC) connector, a full 8-lead microcontroller I/O port, the SPI bus and a 3.3 V power line are made available to a potential peripheral sensor or sensor array that can be placed on a different printed circuit board (PCB).

### 2.4. Power

By all means, the node should not only communicate in a wireless fashion, it should also be powered by an independent power source. Being designed with multiple research applications in mind, the proposed system can be powered by any battery producing a voltage in between 3.3 V and 12 V. To further expand the amount of possible power sources, a micro-USB connector is provided as well, facilitating the use of commercial USB battery packs. The power drawn by the node roughly ranges from 4 mA when in standby mode to 40 mA when sending or receiving LoRa packets. To prevent the RTC from resetting when the battery is changed, a 0.22 F supercapacitor is added, giving the user more time to attach the new battery.

### 2.5. PCB Implementation

**Figure 5:**
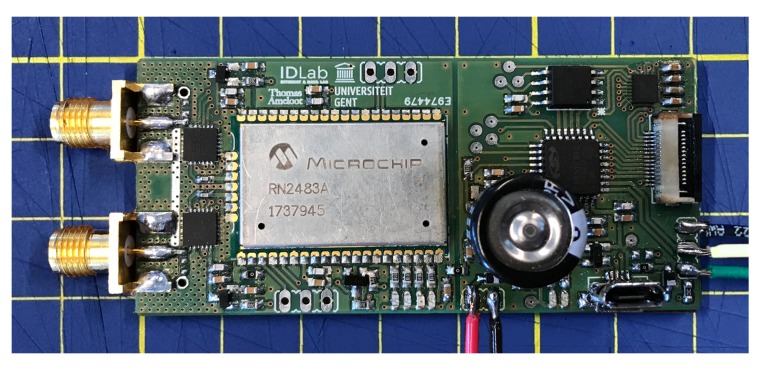
Compact PCB implementation of the wireless sensor node. (One square equals one cm^2^).

Naturally, a compact PCB design is paramount to obtain a significant size reduction when implementing custom hardware instead of using a development board or software radio. To this end, a lot of consideration went into a compact 4-layered PCB layout for this sensor node. The final result is depicted in Figure 5 and measures 70 mm by 32 mm.

## 3. Hardware Verification Setups

Both indoor and outdoor tests were performed to assess the system’s performance. In the following subsections, the setups used for these verification measurements will be discussed in detail.

### 3.1. Outdoor Measurement Setup

**Figure 6:**
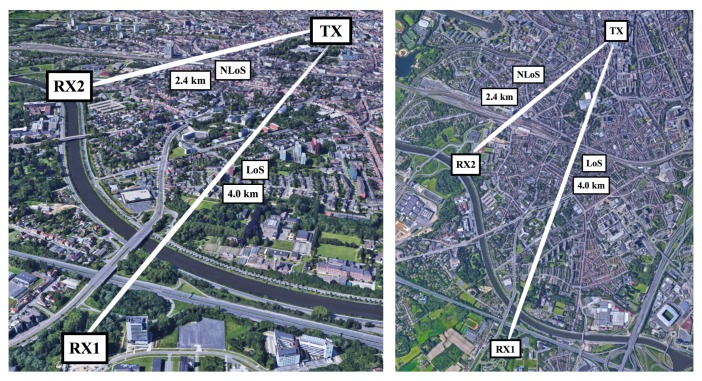
3D and satellite views of the outdoor node locations. Map Data: Google, Landsat/Copernicus.

To perform the outdoor measurements presented in this paper, nodes were placed at three locations in the city of Ghent, Belgium. The exact locations of these sites are marked on Figure 6, which also shows the (sub)urban propagation environment in between the sites. In each of the outdoor experiments, small packets of data were sent from the transmitter location in the center of the city of Ghent to be received by two receivers located near the south of the city.

#### 3.1.1. Transmitter

**Figure 7:**
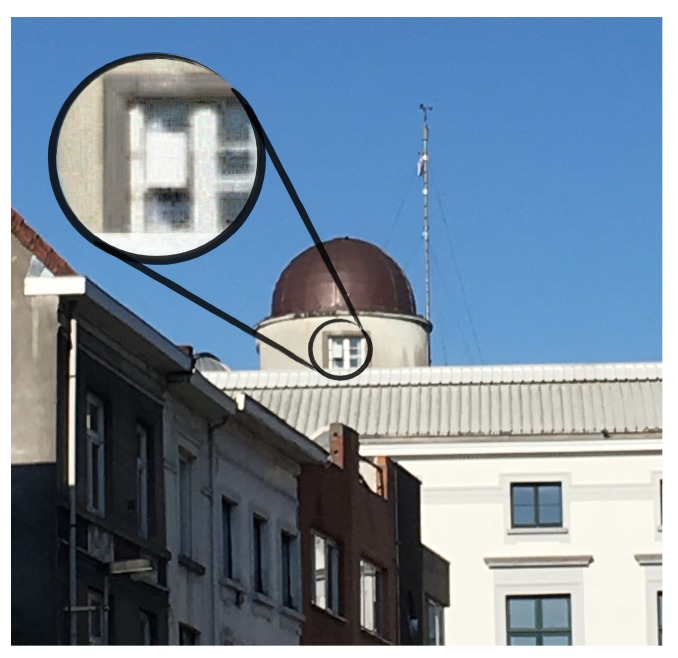
Location of the transmitter at the Armand Pien observatory.

The transmitter (TX) was equipped with two ground plane monopole antennas. For its power supply, the node relied on a heavy-duty battery providing multiple weeks of autonomy. The transmitter node, the antennas and the battery were placed in a waterproof enclosure. As seen in Figure 7, this enclosure was attached to two horizontal iron bars on the outside of a window approximately 20 m above the ground at the south side of a university building, slightly higher than the surrounding houses. To comply with the 1% duty cycle legal restrictions regarding the use of the 434 MHz and 868 MHz ISM-bands, only one packet was transmitted every minute, alternating between both bands. The packet was a 2-byte unsigned integer describing the packet number. A single packet number was, however, used once in each of the two ISM-bands mentioned earlier. Hence, the packet counter increases once every two minutes. The packets were sent at a bitrate of 293 bps and a spreading factor (SF) of 12 was used for the LoRa modulation.

#### 3.1.2. Receivers

The first receiver (RX1) was placed in an office environment at the eleventh floor of a modern building located at the edge of the city of Ghent. The distance from this building to the transmitter is approximately 4.0 km. The line-of-sight (LoS) path includes several urban features such as densely packed houses, a sizable park, a large conference center, a university campus, large apartment buildings, a canal and multiple large roads. The receiver was placed indoors, allowing it to be powered by a power supply. It was connected to similar monopole antennas as used at the transmit side. Yet, the 868 MHz antenna was attached to the outside of the building in order to avoid the signal attenuation by the building’s highly insulating windows, which block the 868 MHz signals in such a way that they could not even be received. Interestingly, 434 MHz signals propagate through these windows considerably better. A second receiver (RX2) was placed indoors, on the fourth floor of a building located closer to the transmitter. For this receiver, the distance to the transmitter equals 2.4 km. At this time, however, the path to the transmitter is non-line-of-sight (NLoS) and includes densely packed houses, a large railway station and a university campus. The hardware setup is equal to the one used for the other receiver (RX1). All of the receivers discussed in this paper store both the received packet numbers as well as the SNR measurements and attenuation settings corresponding to these packets in their flash memory.

### 3.2. Indoor Measurement Setup

**Figure 8:**
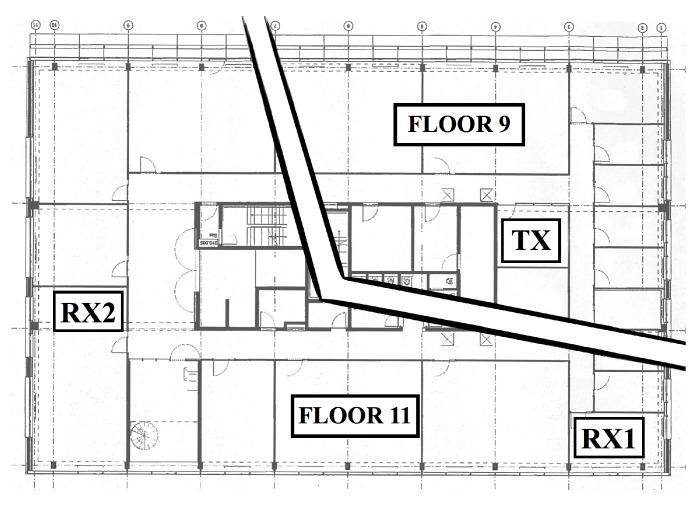
Indoor node locations.

In a second test phase, indoor measurements were performed using a setup in the same building where receiver RX1 was located during the outdoor measurements. Nodes were deployed in three rooms located on two similarly structured floors—the ninth and the eleventh—of the office tower. As seen in Figure 8, the floors of this building all feature a thick concrete core with multiple elevator shafts and stairwells, surrounded by a large number of office spaces.

#### 3.2.1. Transmitter

The transmitter (TX) was placed in a small meeting room on the ninth floor, adjacent to the concrete core of the building. Again, two ground plane monopole antennas were used. At this time, however, the transmitted data packets also included a timestamp and readings of the transmitter’s supply voltage and the ambient temperature.

#### 3.2.2. Receivers

Two receivers were placed in offices on the eleventh floor. The first receiver (RX1) was placed in an office on the same side of the building as the transmitter. Another receiver (RX2) was placed in an office located at the other side of the building. To reach this receiver, radio waves have to propagate through or around the core of the building.

## 4. Measurements and Analysis

According to the measurement setups described in Section 3, multiple sets of test data were gathered. In the following sections, the most interesting data are discussed in detail, but first an outdoor coverage prediction is presented.

### 4.1. Outdoor Received Power Measurements

**Figure 9:**
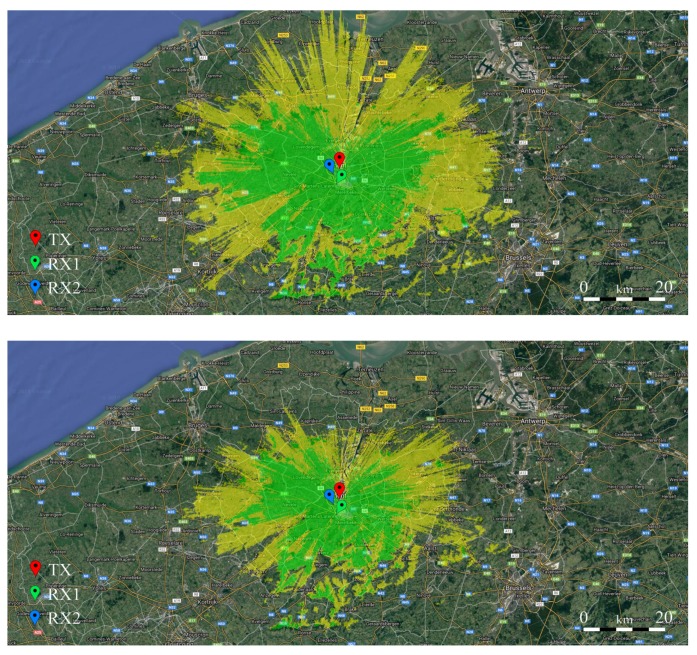
Simulation-based coverage maps for the 434 MHz (**upper map**) and 902 MHz (**lower map**) bands for the transmitter described in Section 3.1. Yellow areas denote the coverage with a reception threshold of −136 dBm and green areas denote the coverage with a link budget that is 10 dB greater than this threshold. Maps generated using Radio Mobile RF by Roger Coudé [21].

To provide more background to the outdoor measurements presented in this paper, coverage maps were generated using the free Radio Mobile RF propagation simulation software (version 03/04/2018) by Roger Coudé [21]. These maps, which can be found in Figure 9, depict the expected coverage for the specific transmitter location discussed earlier for both the 434 MHz and the 868 MHz bands, for transmitting and receiving antennas at 20 m and 13 m above ground, respectively. In these maps, areas colored in yellow denote regions where the expected signal power is equal to the theoretical detection threshold of the system in its default configuration ( −136 dBm), while areas colored in green denote regions where a truly reliable connection could be established (threshold = −126 dBm). Unfortunately, the 868 MHz band is not available for simulation in the tool used to make these predictions, hence the nearby 902 MHz band was used instead, as little difference exists between propagation at 868 MHz and 902 MHz. Note both receivers are situated well within the green area, with a good link budget. Links over a limited distance were selected in order to allow the verification of the correct operation of the automatically adjusting stepped attenuators.

#### 4.1.1. Received Power Measurements at RX1

**Figure 10:**
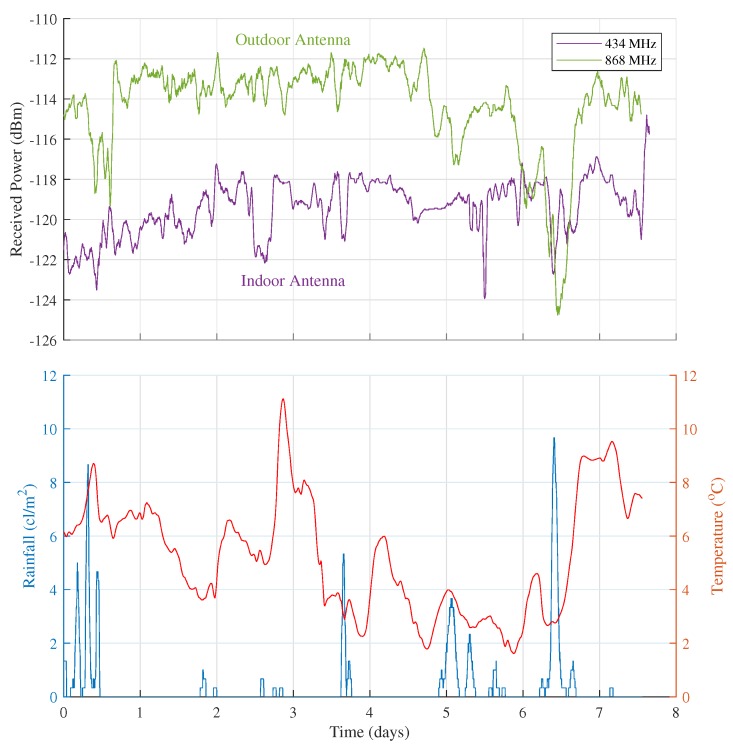
7 days of outdoor received power measurements gathered at RX1 compared to temperature (red curve) and rainfall (blue curve) data.

For a time period of seven days, outdoor measurements were performed according to the measurement setup outlined in Section 3.1. The measurement data recorded by RX1 are presented in the upper half of Figure 10. The data in this part of the graph are filtered by a moving-average window with a size of 30 samples, corresponding to one hour of measurement. The received power fluctuations correspond to standard deviations of 1.29 dB at 434 MHz and 2.09 dB at 868 MHz. Furthermore, according to free space propagation theory, one would expect the average 434 MHz signal to be 6 dB stronger than the average 868 MHz signal. This behavior is not present here, due to the different placement of the antennas (the 434 MHz antenna being placed indoors and the 868 MHz antenna being placed outdoors, as discussed in Section 3.1.2). Moreover, based on the received packet numbers, the packet loss was determined. For this test, this loss adds up to 0.61% for the 434 MHz band and 6.11% for the 868 MHz band. The packet loss in the latter band is higher because of collisions with packets from other transmitters. A more detailed analysis indeed revealed a much higher occupation of the 868 MHz band, when compared to the 434 MHz band.

To investigate the origin of the sizable drop in the received power of the 868 MHz signal on day 6, temperature and rainfall data was provided by the Armand Pien weather observatory. These data are presented in the lower half of Figure 10. On day 6, a sizable peak in rainfall was observed which coincides directly with the signal level dip. A slightly lower amount of rainfall was observed on the first day of measurement (day 0), resulting in a smaller, but still measurable drop in signal level on that day. These signal dips are however not present in the 434 MHz data, which were measured with an indoor antenna. The origin of this feature is most probably to be found in the antenna placement. With the 868 MHz antenna being placed outdoors, the humidity on this antenna is expected to have changed its behavior in such a way that a mismatch occurred, causing the drop in signal. Consequently, for outdoor operation more weatherproof antennas should be selected. The temperature on the other hand did not influence the measured data in any discernible way as temperature fluctuations were too limited to have a significant impact on the receiver noise. Finally, it has to be noted that because of the relatively low received signal values, this receiver’s attenuators remained at 0 dB during this experiment.

#### 4.1.2. Received Power Measurements at RX2

**Figure 11:**
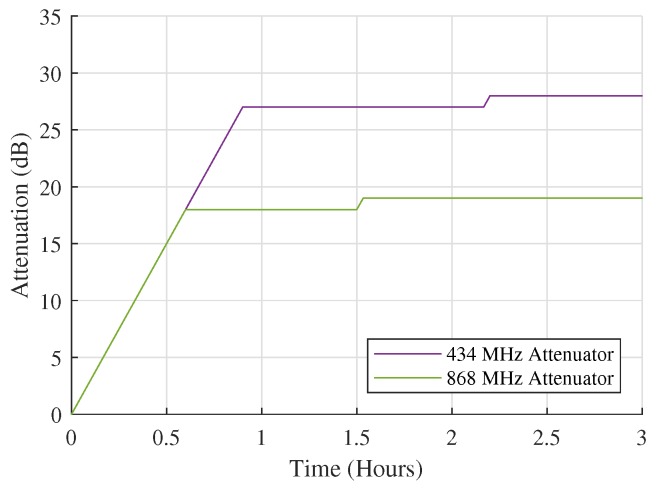
Attenuator startup behavior at outdoor receiver RX2. The slow response is a result of legal restrictions, but also prevents the system from reacting to sudden, temporary effects.

To verify the correct operation of these attenuators and the dynamic attenuation adjustment algorithm that controls them, similar measurements were performed by receiver RX2. As this receiver was located closer to the transmitter, in a building with older, conventional—not highly insulating—glass windows, it was expected that the attenuation settings would be altered immediately after receiving the first packets and would continue to change until the detector was no longer saturated. Figure 11 shows the actual startup behavior of the node, which indeed corresponds to this expected behavior. The slope in the graph corresponds to an increase in attenuation of 30 dB/h, which is equal to 1 dB every two minutes. This can of course be explained by legal restrictions only allowing the transmitter to send one packet every two minutes in each band. The slow response was deliberately conserved because we do not want the system to overreact to sudden changes caused by temporary effects such as potential interference. It should also be noted that the attenuation settings are only altered when receiving packets with the right ID tag, originating from our own transmitter. Consequently, potential strong signals from unknown LoRa sources do not influence the attenuation settings.

**Figure 12:**
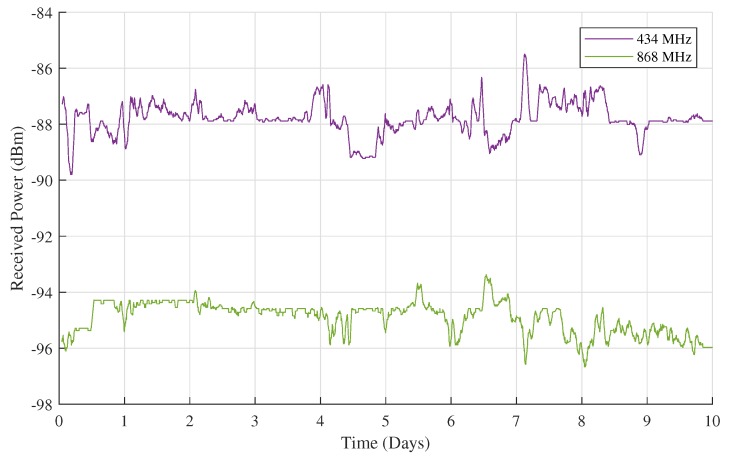
10 days of outdoor received power measurements gathered at RX2.

The received power measurements gathered during this ten-day test are presented in Figure 12. Again, an averaging window of one hour is used. With standard deviations of 0.61 dB for the raw 434 MHz data and 0.64 dB for the raw 868 MHz data, these measurements show a lot less fluctuation than the ones made by receiver RX1. This could be attributed to the smaller distance to the transmitter and the resulting larger signal-to-interference ratio (SIR). Also in contrast to the data presented in Section 4.1, this time, the theoretically expected signal power difference of 6 dB between the 434 MHz band and the 868 MHz band is clearly visible. In conclusion, the packet loss for this second test is calculated, adding up to a mere 1.17% for the 434 MHz band and 1.6% for the 868 MHz band. This second packet loss value is a lot smaller than the packet loss measured for the 868 MHz band at RX1 thanks to the lower height of RX2 and the smaller number of nearby LoRa sources at RX2.

### 4.2. Indoor Signal Level Measurements

**Figure 13:**
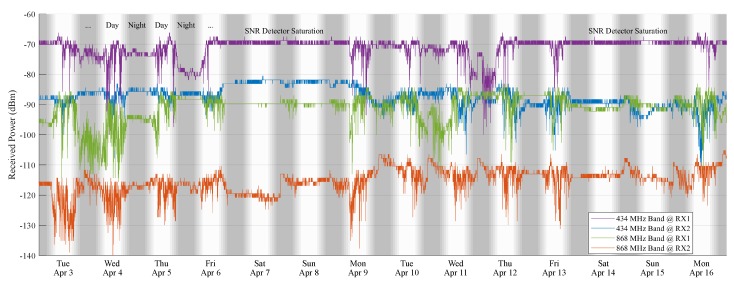
14 days of indoor received power measurements.

Finally, the indoor setup described in Section 3.2 was considered to gather a third set of signal measurements. These data are presented in Figure 13. They document half a month of indoor LoRa channel measurements performed in the first half of April 2018. The first observation we make on this graph is that the average received powers of the 434 MHz signals ($\mu_{434,RX1}$ = −70.9 dBm and $\mu_{434,RX2}$ = 88.2 dBm-all averages were calculated using dBm values) are considerably larger than those of the corresponding 868 MHz signals ($\mu_{868,RX1}$ = −91.7 dBm and $\mu_{868,RX2}$ = −116.0 dBm). Hence, it can be concluded that using the 434 MHz ISM-band is indeed a much better option for indoor LoRa communication than using the 868 MHz ISM-band.

Considering the signal fluctuations, a clear distinction can be made between data gathered when the building was empty and data gathered when it wasn’t. During weekend days, the received signals are much more stable than on week days. Furthermore, these fluctuations mostly disappear by the end of the work day to reappear the next morning. This indicates that the presence of people has a clear and measurable influence on the stability of the LoRa channel. To properly describe this behavior, the mean values and standard deviations of the received powers are calculated for intervals of 8 h during the day (9 a.m.–5 p.m.) and during the night (9 p.m.–5 a.m.). To clarify this, a subset of these descriptive statistics can be found in Table 1, describing the second week of indoor measurements at RX1. Now, the averages of the mean values (denoted by $\bar{\mu }$) of the subsets of signal data that describe the channel performance when there are people in the building (by day, on working days) and those subsets of data that describe the channel performance when people are not around (at night and in the weekends) are calculated for both bands and both receivers. Moreover, the averages of the standard deviations (denoted by $\bar{\sigma }$) of the received powers are calculated for the same subsets (DAY vs. NIGHT, RX1 vs. RX2 & 434 vs. 868). These values can be found in Table 2. Although there is no significant change in mean received signal power, for both receivers RX1 and RX2 and both ISM-bands, there is a clear difference between the standard deviations of the power levels measured when people are around and when they are not. This confirms the claim stated earlier that the channel is less stable when people are present in the building. Signal levels remained well above the detection limit of the LoRa unit configured with the default parameters for the 434 MHz band for both receivers, whereas this was not the case for receiver 2 in the 868 MHz band.

As a side note to Figure 13, it should be mentioned that due to the small distance between the transmitter and RX1 and the use of unidirectional LoRa-communication for this test, the detector of RX1 is often saturated when measuring the SNR of packets received in the 434 MHz band. Nevertheless, during week days, this detector comes out of saturation, which further establishes the influence of the presence of people on the quality of the LoRa link. This saturation behavior could be mitigated by signaling this back to the transmitter using bidirectional LoRa-communication. This way, the transmitter could also increase its attenuation settings. Naturally, as was discussed in Section 2.2, this may increase the packet loss of other receivers, illustrating the trade-off between receiving more low-power packets and detecting higher received power values.

## 5. Conclusions

An autonomous LoRa-compatible wireless sensor node for the IoT was designed, fabricated, calibrated and tested. This node is not only unique in its ability to perform channel characterization in both the 434 MHz and 868 MHz ISM-bands, it can also accurately measure received power levels over a large dynamic range. A feat which is possible thanks to the stepped attenuators implemented in this design and the calibration of the unit performed in the lab.

Furthermore, several design choices (such as the addition of a real-time clock with a dedicated interrupt line and a supercapacitor buffering the supply voltage) were made to increase the autonomy of the node, enabling prolonged measurement campaigns to take place. Moreover, both the incorporation of various other peripheral components and the fact that this custom hardware is significantly smaller than a development board or software radio show that this system qualifies for a very broad range of LoRa research applications.

The correct operation of the node was successfully validated by performing both indoor and outdoor test measurements. In these measurement campaigns, the expected superiority of the 434 MHz band for long-range communication links was repeatedly confirmed. Moreover, with measured received power values ranging from −140 dBm to −70 dBm, the need for and usefulness of an enhanced dynamic range for channel measurements was clearly illustrated.

The outdoor tests have also provided insight into the difference in link quality between the 434 MHz and 868 MHz LoRa channels. With an additional distance of 1.6 km to the transmitter, the standard deviation of the SNR of the packets received by receiver RX1 exhibited an increase of 1.29 dB for the 434 MHz band and 3.98 dB for the 868 MHz band when compared to the SNR of the packets received by the more nearby RX2. When comparing rainfall and temperature data to the signals measured outdoors, a correlation was found between a sudden drop in signal and an episode of rainfall. This indicated that the behavior of the antenna, which was placed outside of the building, had changed substantially due to the water present on it. No correlation was found between the outdoor temperature fluctuation and the measured signal levels.

Indoor measurements indicated that the presence of people in a building also has a measurable influence on the quality of the LoRa link. When considering the 434 MHz band, the average standard deviations of the received powers measured by day differ from these measured by night or in the weekend by approximately 2 dB for both indoor receivers. For the 868 MHz band, these differences amount to approximately 3 dB for both receivers.

In conclusion, the system successfully recorded several propagation characteristics in both the 434 MHz and 868 MHz ISM-bands over a wider dynamic range than achievable with off-the-shelf LoRa units.

## Figures and Tables

**Table 1:** Statistics describing the second week of indoor power measurements gathered at RX1. (DAY: 9 a.m.–5 p.m. & NIGHT: 9 p.m.–5 a.m.).

	434				868			
	DAY		NIGHT		DAY		NIGHT	
	μ	σ	μ	σ	μ	σ	μ	σ
Monday 9 April	−72.4	4.0	−70.3	0.8	−89.5	4.1	−91.3	1.9
Tuesday 10 April	−70.9	2.3	−71.9	1.2	−88.9	2.8	−96.6	4.4
Wednesday 11 April	−71.1	2.4	−79.8	5.3	−88.7	4.0	−86.9	0.7
Thursday 12 April	−70.1	2.7	−69.8	0.6	−89.3	5.2	−87.0	0.1
Friday 13 April	−71.9	3.4	−69.5	0.6	−89.2	4.1	−90.7	0.6
Saturday 14 April	−69.7	0.6	−69.4	0.6	−91.3	0.6	−88.8	1.4
Sunday 15 April	−69.2	0.5	−69.4	0.6	−90.7	0.8	−89.2	1.6

All values expressed in dBm.

**Table 2:** Average values of the descriptive statistics describing subsets of indoor power measurements. (DAY: 9 a.m.–5 p.m. & NIGHT: 9 p.m.–5 a.m.).

	434				868			
	DAY		NIGHT		DAY		NIGHT	
	$\bar{\mu }$	$\bar{\sigma }$	$\bar{\mu }$	$\bar{\sigma }$	$\bar{\mu }$	$\bar{\sigma }$	$\bar{\mu }$	$\bar{\sigma }$
RX1	−71.11	2.998	−70.75	0.8242	−90.82	4.296	−92.52	1.086
RX2	−88.84	2.745	−87.51	0.7685	−116.5	4.052	−115.4	1.349

All values expressed in dBm.

## Abbreviations

The following abbreviations are used in this manuscript:

WSN	Wireless Sensor Network
IoT	Internet of Things
CSS	Chirp Spread Spectrum
SNR	Signal-to-Noise Ratio
ISM-band	Industrial, Scientific and Medical Radio Band
SMBus	System Management Bus
I^2^C	Inter-Integrated Circuit
UART	Universal Asynchronous Receiver-Transmitter
SPI	Serial Peripheral Interface
I/O	Input/Output
USB	Universal Serial Bus
RF	Radio Frequency
LoRaWAN	LoRa Wide Area Network
SF	Spreading Factor
RBW	Receiver Bandwidth
FSK	Frequency Shift Keying
GFSK	Gaussian Frequency Shift Keying
IMU	Inertial Measurement Unit
RTC	Real-Time Clock
FFC	Flat Flexible Cable
FPC	Printed Circuit Cable
PCB	Printed Circuit Board
TX	Transmitter
RX1	Receiver 1
RX2	Receiver 2
LoS	Line-of-Sight
NLoS	Non-Line-of-Sight
SIR	Signal-to-Interference Ratio
